# The role of priors in Bayesian models of perception

**DOI:** 10.3389/fncom.2013.00025

**Published:** 2013-04-03

**Authors:** Christoph Teufel, Naresh Subramaniam, Paul C. Fletcher

**Affiliations:** ^1^Brain Mapping Unit, Department of Psychiatry, University of CambridgeCambridge, UK; ^2^Cambridge and Peterborough Foundation TrustCambridge, UK

In a recent opinion article, Pellicano and Burr (2012) speculate about how a Bayesian architecture might explain many features of autism ranging from stereotypical movement to atypical phenomenological experience. We share the view of other commentators on this paper (Brock, 2012; Friston et al., 2013; Van Boxtel and Lu, 2013) that applying computational methods to psychiatric disorders is valuable (Montague et al., 2012). However, we argue that in this instance there are fundamental technical and conceptual problems which must be addressed if such a perspective is to become useful.

**Figure 1:**
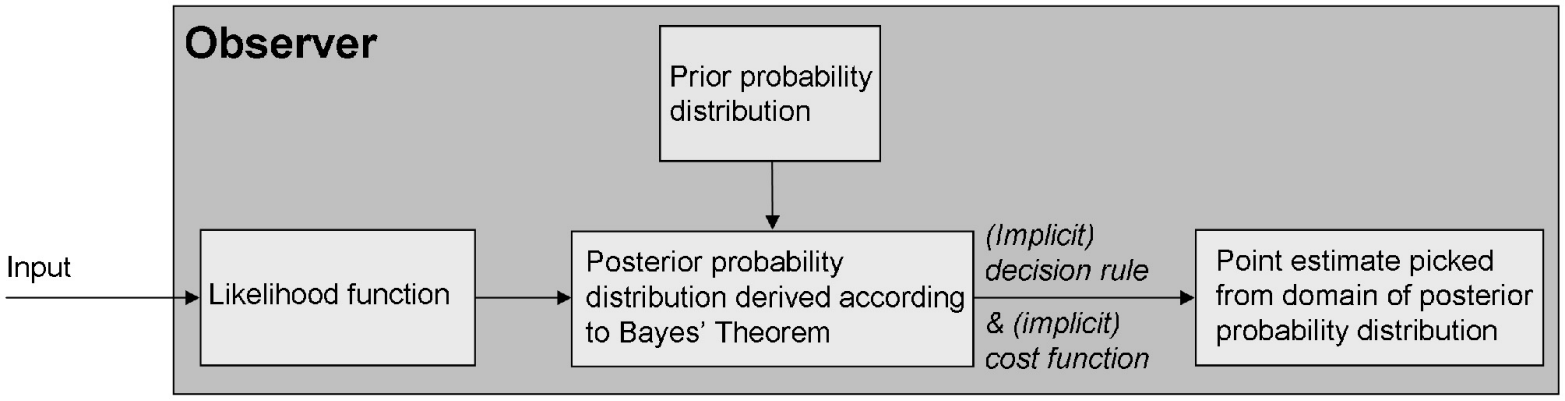
**The standard Bayesian observer model (Geisler and Kersten, 2002) embodies the assumption that perception is based on two sources of information: prior beliefs about the state of the world are updated by incoming sensory information; perceptual decisions are then based on the updated beliefs.** For the purpose of modeling, the psychological concepts of belief, sensory processing, perception, and decision are mapped onto mathematical concepts derived from probability theory: respectively, these are the prior, the likelihood function, the posterior, and the cost function. Specifically, the observer's belief about the statistical regularities of the world is modeled by a probability distribution (the prior); the observer's noisy sensory processing is reflected in a measurement distribution, which specifies a likelihood function; the combination of prior and likelihood according to Bayes' theorem models the combination of belief and sensory information; this results in an *a posteriori* probability distribution (the posterior), representing the space on which the perceptual decision is made. One value picked from the domain of this distribution according to an implicit decision rule, determined by gains and losses specified in a cost function, represents the ultimate percept of the observer.

Based on the Bayesian observer model (Figure 1), Pellicano and Burr speculate that perceptual abnormalities in autism can be explained by differences in how beliefs about the world are formed, or combined with sensory information, and that sensory processing itself is unaffected (although, confusingly, they also speak of *sensory* atypicalities in autism). In computational terms, the authors are suggesting that the likelihood function is unaltered in autism and that the posterior is atypical either because of differences in the prior, or because of the way in which prior and likelihood are combined. The latter statement is problematic because within the framework of probability theory, the combination of these two components is fixed as determined by Bayes' theorem: they are multiplied. Put simply, a mathematically consistent Bayesian model cannot accommodate a perceptual abnormality in autism that is due to the way in which belief and sensory information, i.e., prior and likelihood, are *combined*. Furthermore, if sensory processing is mathematically represented as a likelihood function (as it typically is within Bayesian models), then changes in the prior cannot lead to changes in sensation, as the authors claim (they can only lead to changes in perception).

A second problem is the lack of justification for the view that autistic atypicalities in perception exclusively relate to differences in the prior. Typically, Bayesian models start with three unknowns—prior, likelihood, and cost function—and atypicalities might therefore be due to any of these components (also see Brock, 2012). This pertains to both the family of distributions or functions, which these components come from, and their specific parameter values. Given this flexibility of Bayesian models, meaningful results can only be obtained if the uncertainty about the three unknowns is theoretically or empirically constrained in a principled manner.

Third, the center piece of Pellicano and Burr's proposal, emphasized in the title and the abstract, is the assertion that due to a “broadening” of the prior, autistic perception is more accurate in the sense that it is closer to physical reality. We believe that this idea is fundamentally misguided and deserves immediate attention because it turns not only Bayesian modeling of perception but Bayesian statistics as a whole on its head. Taken to its logical extreme, the authors' claim is that perception unrefined by prior beliefs about the world is the best assessor of reality. However, the reason why Bayesian statistics have been so generally successful is that combining knowledge of the statistical regularities of the world in the form of a prior with a noisy measurement provides a more accurate estimate of the veridical value than solely relying on measurement (Chernoff and Lincoln, 1959; Berger, 1985; Young and Smith, 2005). This is why Bayesian statistics were adopted in modeling of perception; they offer an optimal solution to exactly the problem that any perceptual system faces—inferring the hidden causes of its sensory measurements under uncertainty (Geisler and Kersten, 2002)—and can therefore serve as a benchmark. Only if the knowledge represented in the prior systematically differs from the statistics of the current situation, does its incorporation result in a less accurate (or less “real”) perceptual system. Within the Bayesian framework, this is how illusions are explained because observers apply natural-image statistics to an artificial laboratory situation (Weiss et al., 2002). Yet, exactly the same priors provide more accuracy in the natural world than would measurement alone. Put differently, under those circumstances for which the prior has developed—perception under naturalistic circumstances—, a “hypo-prior” would do the opposite to what the authors claim, namely, it would lead to less accurate perception. Illusions thus do not illustrate that priors *per se* render perception less accurate; rather, it is the application of the *wrong* prior that leads to the illusory percept.

While one might envisage that “broader” priors lead to a perception that is closer to sensory input, this does *not* mean that the observer would perceive the world as more “real.” In order to regulate its interactions with the environment, an observer needs appropriate information about the states of the world. Yet, there is no direct access to this information but only to sensory stimulation caused by the environment. This poses a computational problem because sensory information is noisy and inherently ambiguous. On its own, it rarely suffices to uniquely specify the environment. The Bayesian framework provides an optimal solution to this challenge by resorting to an additional source of information—prior knowledge of the world's regularities—that allows disambiguation of the incoming sensory information. Put simply, a perceptual system that refines sensory information by prior knowledge provides a better estimate of real but hidden causes than perception that is based on the ambiguous sensory information on its own because the former system exploits all the relevant information available.

Pellicano and Burr list a large number of phenomena that, they argue, can be explained by weaker priors in autistic individuals. It is therefore not surprising that some of these examples such as impaired performance on some perceptual tasks are, at least theoretically, consistent with a Bayesian framework. The possible consistency, however, would not rectify the problems that, we believe, attend the central conceptual tenet of the paper. Moreover, it is noteworthy that some of the examples are vulnerable to empirical criticism. The primary phenomenon that the authors cite in support of their hypothesis—a reduced susceptibility in autism to some illusions (Happé, 1996)—has not been replicated more recently (Hoy et al., 2004; Mitchell and Ropar, 2004; Milne and Scope, 2008; Simmons et al., 2009). This issue seems to pose a critical challenge to the general claims expressed in the article.

We agree that Bayesian models provide a principled way of thinking about visual perception in autism. However, much work remains to be done in providing full mathematical formalizations of specific, isolated symptoms. Once such data are available for a range of abnormalities, it will be useful to revisit the challenge of providing an overarching computational architecture of autistic perception.
